# Assessment of the Italian Version of the Internet Disorder Scale (IDS-15)

**DOI:** 10.1007/s11469-017-9823-2

**Published:** 2017-10-30

**Authors:** Lucia Monacis, Maria Sinatra, Mark D. Griffiths, Valeria de Palo

**Affiliations:** 1 Department of Humanities, University of Foggia, via Arpi, 175, 71100 Foggia, Italy; 2 Department of Educational Sciences, Psychology, Communication, University of Bari Aldo Moro, Bari, Italy; 3 International Gaming Research Unit, Division of Psychology, Nottingham Trent University, Nottingham, UK; 4 Department of Humanities, University of Foggia, Foggia, Italy

**Keywords:** IDS-15, Internet disorder scale, Psychometric properties, Latent profile analyses, Behavioral addiction, Internet addiction

## Abstract

Much research has focused on the validation of psychometric tools assessing Internet addiction. One of the newest measures is the Internet Disorder Scale (IDS-15) based on the modified IGD criteria outlined in the Diagnostic and Statistical Manual of Mental Disorders (DSM-5). This study aimed at investigating the psychometric properties of the Italian version of the IDS-15 by examining the construct and the concurrent and the criterion-related validity and by identifying the taxonomy and the patterns of Internet users. A sample of 471 participants (*M*_age_ = 24.72 years, SD = 8.66; 256 males) was recruited from secondary schools, universities, and gaming halls. Confirmatory factor analyses supported the four-dimensional second-order structure and the three levels of the measurement invariance across gender. The reliability and the validity of the scale were confirmed, and the LPAs provided four classes of Internet users on the basis of the scores obtained in all four dimensions of the scale. The psychometric robustness of the Italian version of the IDS-15 was clearly demonstrated. Cross-cultural research should expand and generalize the present findings.

The Internet and its related technologies have revolutionized the way in which the world operates including individuals’ lifestyles. Although the many positive applications of digital technologies have been recorded in (mental) health, education, and leisure, the large increase of online involvement has conversely led researchers to examine in-depth negative consequences based on similarities with substance-related addictions, such as psychopathological symptoms, subjective distress, health problems, mood modification, conflict, and relapse (see Griffiths 2005; Kuss et al. 2014 for reviews).

Over the last few years—and often using Griffiths’ (2005) biopsychological components model of addiction (i.e., cognitive salience, mood modification, tolerance, withdrawal, conflict, and relapse)—empirical studies have been carried out in the area of technological addictions, leading to the inclusion of the Internet Gaming Disorder (IGD) in the Section III (“Emerging measures and models”) of the new edition of the latest (fifth) edition of the Diagnostic and Statistical Manual of Mental Disorders (DSM-5; American Psychiatric Association (APA) 2013). This has led to the growing interest of researchers in the development and validation of new standardized psychometric tools for the assessment of various technological behavioral addictions. There have been calls for a shared nomenclature of the IGD concept and its standardized psychometric measure (Griffiths et al. 2014). This led to development of the nine-item Internet Gaming Disorder Scale short-form (IGDS9-SF) based on the DSM-5 criteria (Pontes and Griffiths 2015a) to help foster scientific consensus regarding the overuse of digital gaming technologies and to aid prevention policies and treatment. The robust psychometric validity and reliability of the IGDS9-SF has been also been demonstrated in international contexts including translations into Spanish, Portuguese, Italian, Slovenian, and Persian (Fuster et al. 2016; Monacis et al. 2016; Pontes and Griffiths 2016; Pontes et al. 2016; Stavropoulos et al. 2017; Wu et al. 2017).

Particular attention has also been paid to the conceptualization of Internet addiction (IA), which has been defined as an umbrella term comprising a wide range of problematic online activities (e.g., online gaming, online gambling, online sex, social networking) (Kuss and Billieux 2017) and is characterized by excessive preoccupation and/or urges to use the Internet and poorly controlled behaviors on the Internet that imply impairment or distress in many life domains (Weinstein et al. 2014). Despite empirical research showing that IA is a serious condition associated with psychosocial factors for a small minority of individuals (Cerniglia et al. 2017; Kuss et al. 2014; Pontes et al. 2015), such characterization has led to an inadequate psychometric assessment of IA (Király et al. 2014). In light of the critical methodological issues such as the absence of a gold standard to assess the IA, Cho et al. (2014) developed a self-diagnostic IA scale based on the diagnosis criteria for IGD. However, some limitations emerged in the factor structure due to the lack of correspondence of the factors with each criterion suggested in the DMS-5, leading Pontes and Griffiths (2017) to develop the Internet Disorder Scale (IDS-15) by applying the modified IGD criteria as outlined in the DSM-5 (APA 2013).

Because this new instrument showed evidence of robust psychometric properties and met most of the criteria suggested by Koronczai et al. (2011) for a suitable measure (comprehensiveness, brevity, reliability and validity, reliability and validity across different age groups, cross-cultural reliability and validity, and validation on clinical samples), the aim of the present study was to (i) investigate the psychometric properties of the Italian version of the IDS-15 by examining its construct validity (i.e., the factorial structure and the measurement invariance (MI) across gender groups), (ii) analyze the associations between the translated version and other related constructs in order to assess the concurrent and criterion validity of scores on the IDS-15, and (iii) carry out a latent profile analysis (LPA) in order to identify the taxonomy and the patterns of Internet users, as well as their potential risk of IA.

## Method

### Participants and Procedure

The sample comprised 471 participants (*M*_age_ = 24.72 years, SD = 8.66; 256 males) recruited from Italian secondary schools, universities, and gaming halls. Participants were invited to fill out a self-report questionnaire which took approximately 15 minutes to complete. Sixteen cases were removed after cleaning the dataset. The final sample comprised 455 respondents of which 79.6% were high school graduates, 67.9% were non-smokers, and 27.3% spent from 2 to 4 hours per day on the Internet. The mean age of Internet use initiation was 15.99 years (SD = 7.81). The scale was translated from English into Italian separately by the Italian authors of the present study. The translated versions were compared and back-translated into a single English version by a native speaker following the protocol set out by Beaton et al. (2000). No discrepancies between the two versions emerged.

### Measures

#### Socio-Demographics

The survey included questions concerning gender, age, educational level, daily Internet usage (average daily hours spent on the Internet for leisure purposes), age of Internet use initiation (age participant remembers first using the Internet), and cigarette usage (whether the participant smoked more than three cigarettes per day).

#### Internet Disorder Scale (IDS-15)

The IDS-15 (Pontes and Griffiths 2017) assesses the severity of IA and the impact of its effects by focusing upon users’ online leisure activity (i.e., excluding academic and/or occupational Internet use) from any device with Internet access over the past year. The 15 items are rated on a 5-point Likert scale (from 1 = *strongly disagree* to 5 = *strongly agree*) and assess four distinct IA-related domains: (i) *escapism and dysfunctional emotional coping*, (ii) *withdrawal symptoms*, (iii) *impairments and dysfunctional self-regulation*, and (iv) *dysfunctional Internet-related self-control*. The total score is obtained by summing up participants’ responses and can range from 15 to 75, with higher scores being an indication of higher degrees of IA.

#### Internet Gaming Disorder Scale-Short Form (IGDS9-SF)

The Italian version of the IGDS9-SF (Monacis et al. 2016) assesses the severity of IGD and the detrimental effects of online and/or offline gaming activities occurring over the past year. The scale comprises nine items answered on a 5-point Likert scale ranging from 1 (never) to 5 (very often). Examples of items are “Have you lost interest in previous hobbies and other entertainment activities as a result of your engagement with the game?” and “Do you feel more irritability, anxiety or even sadness when you try to either reduce or stop your gaming activity?” A higher score indicates a higher degree of gaming disorder. In the present study, the scale had excellent reliability (Cronbach’s *α* = .947).

#### Social Media Addiction Scale (BSMAS)

The Italian version of the BSMAS (Monacis et al. 2017a) evaluates experiences in the use of social media over the past year. It comprises six items rated on a 5-point Likert scale (from 1 = *very rarely* to 5 = *very often*). Examples of items are “How often during the last year have you spent a lot of time thinking about social media or planning use of social media?” and “How often during the last year have you used social media so much that it has had a negative impact on your job/studies?” In the present study, the internal consistency of the scale was very good (Cronbach’s *α* = .833).

### Ethics

The study procedures were carried out in accordance with the Declaration of Helsinki. The investigation was approved by the research team University’s Institutional Review Board. Permission was required from heads and deans to conduct the research study at the school/institution. Written informed consent was obtained from students over 18 years of age, whereas parents or legal guardians provided written consent for students aged under 18 years to participate.

### Statistical Analysis

The cleaning of the dataset involved the inspection of cases with missing values and the checking of the univariate and multivariate normality of all items of the IDS-15. As for the univariate normality, no item showed absolute values of skewness greater than 2.0 or values of kurtosis greater than 7.0 (Kim 2013). The univariate outliers were also identified using the graphic approach (inspection of a boxplot). Although a total of 10 sets of answers proved to be distant from the others, they were not removed from the analyses because their higher scores on the IDS-15 contained potentially valuable information about the phenomenon. The multivariate outliers were inspected using Mahalanobis distances and the critical value for each case based on the chi-square distribution values. The procedure yielded 16 cases that were then removed from subsequent analyses.

Substantive analyses comprised (i) descriptive statistics (means, standard deviations, frequencies); (ii) independent samples *t* test to verify gender effects on the scores of the variables taken into account; (iii) assessment of the construct validity of the IDS-15 that involved factorial validity (via confirmatory factor analysis [CFA]), measurement invariance (MI) (using multigroup CFAs across gender), and convergent and discriminant validity (based on the comparison of the average variance extracted [AVE] coefficients and the maximum shared squared variance [MSV] of each latent variable); (iv) reliability coefficients and indicators of internal consistency (i.e., Cronbach’s alpha, composite reliability [CR], and factor determinacies [FD]; (v) the latent profile analysis (LPA) to identify different groups of Internet users on the basis of their responses to the four subscales of the IDS-15 (the number of latent classes was established following Pontes and Griffiths’ (2017) study, i.e., two to four classes, and was determined using (a) the Akaike Information Criteria (AIC), the Bayesian Information Criteria (BIC), and the Sample-Size Adjusted BIC (SSABIC) with lower values indicating more parsimonious models; (b) the Lo-Mendell-Rubin Adjusted Likelihood Ratio test (L-M-R test) with a significant *p* value (< .05)); and (vi) criterion-related validity (i.e., bootstrapped correlation with bias-corrected accelerated 95% confidence intervals between the IDS-15 overall scores and the chosen criteria).

## Results

### Descriptive Statistics

Table 1 summarizes the means and standard deviations (SDs) of the IDS-15 (for total score and for each dimension), BSMAS, and IGDS9-SF scores.

**Table 1:** Mean score and standard deviation of IDS and its four dimensions, BSMAS and IGDS for total sample and gender group

	Mean (SD)		
	Total sample	Females	Males
IDS-15	35.95 (11.00)	35.62 (10.37)	36.24 (11.53)
EDEC	2.71 (.829)	2.66 (.78)	2.75 (.87)
WS	2.20 (.943)	2.20 (.93)	2.21 (.95)
IDSR	2.24 (.870)	2.27 (.82)	2.21 (.91)
DISC	2.45 (1.050)	2.36 (.96)	2.53 (1.13)
BSMAS	13.69 (5.091)	14.36 (4.76)	13.13 (5.30)
IGDS9-SF	14.49 (7.463)	12.32 (5.75)	16.31 (8.22)

*IDS-15* Internet Disorder Scale, *EDEC* escapism and dysfunctional emotional coping, *WS* withdrawal symptoms, *IDSR* impairments and dysfunctional self-regulation, *DISC* dysfunctional Internet-related self-control

### Independent Sample *t* Tests

Significant gender differences emerged in the scores of BSMAS, *t*_(453)_ = − 2.58, *p* < .05, and IGDS9-SF, *t*_(438,962)_ = 6.06, *p* < .001. More specifically, females obtained higher scores in BSMAS, whereas males obtained higher scores in IGDS9-SF (Table 1). No differences emerged on total IDS scores or its dimensions.

### Construct Validity

**Fig. 1:**
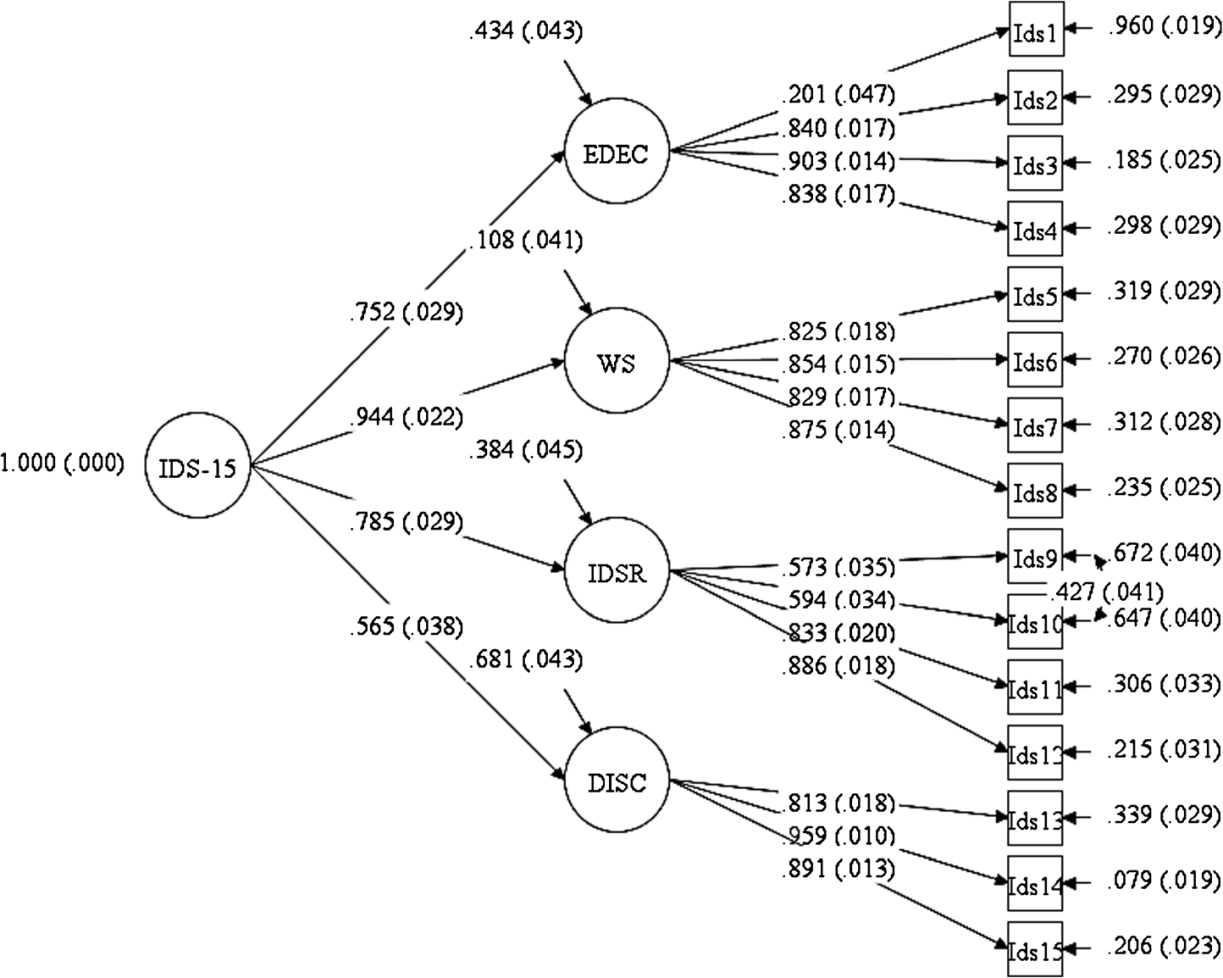
Graphical representation of Model 3

In order to test the factorial structure of the IDS-15, CFAs were performed with the Mean and Variance Adjusted Maximum Likelihood (MLMV) method. The chi-squared (*χ* ^2^) and its degree of freedom (test values associated with *p* > .05), the Comparative-of-Fit Index (CFI; values ≥ .90), the Root Mean Square Error of Approximation (RMSEA; values close to .06) plus its 90% confidence interval (CI), and the Standardized Root Mean Square Residuals (SRMR; values ≤ .08) were accepted as indicators of good fit. Model 1 with a unidimensional structure showed inadequate fit values. Model 2 with four factors yielded adequate fits, although the RMSEA value (.07) was higher than the expected threshold level. A careful inspection of modification index values indicated that items 9 and 10 should be correlated. Thus, Model 2 with a four-factor solution and two correlated items provided better fits. Model 3 with a second-order structure indicated fit values equal to Model 2, although the AIC and BIC values indicated better fit indices for Model 3 (Table 2). Figure 1 depicts the graphical representation of Model 3.

**Table 2:** Goodness-of-fit indices of the four CFAs

Models	*χ* ^2^	*df*	*p* value	RMSEA	90% CI	CFI	SRMR	AIC	BIC
Model 1	1663.800	90	.000	.196	.188–.204	.658	.104	17,484.703	17,670.116
Model 2	186.956	83	.000	.052	.042–0.063	. 977	.047	16,021.859	16,236.115
Model 3	189.440	85	.000	.052	.042–0.062	. 977	.047	16,020.343	16,226.357

*Model 1* unidimensional first order, *Model 2* four-dimensional first order with two correlated items (item 9 and item 10), *Model 3* four-dimensional second-order structure with two correlated items (item 9 and item 10)

Measurement invariance of Model 3 across gender was evaluated using MLMV estimation to test for configural, metric, and scalar invariance. The classical approach based on the *χ* ^2^ difference (Δ*χ* ^2^) test and the comparison of two nested models using cut-off values of ∆CFI < .01 and ∆RMSEA < .015 for metric and scalar invariance were considered (Table 3).

**Table 3:** Measurement invariance by gender

	*χ* ^2^	*df*	Δ*χ* ^2^	Δ*df*	Sig.	TLI	CFI	RMSEA	ΔCFI	ΔRMSEA
Females	110.997	85	–	–	–	.975	.980	.038	–	–
Males	127.253	85	–	–	–	.972	.977	.045	–	–
Configural	237.602	170	–	–	–	.973	.978	.042	–	–
Metric	249.997	181	12.40	11	.335	.974	.978	.041	.000	.001
Scalar	261.182	192	11.19	11	.428	.976	.978	.040	.000	.001

With regard to convergent and discriminant validity, AVE values greater or equal to .50 and MSV values smaller than AVE indicate adequate validity (Table 4).

**Table 4:** Reliability coefficients

	*α*	CR	FD	AVE	MSV
IDS_15	.915	.852	.935	.598	–
IDS_EDEC	.757	.816	.954	.565	.514
IDS_WS	.909	.909	.961	.715	.544
IDS_IDSR	.835	.819	.941	.540	.231
IDS_DISC	.916	.919	.973	.791	.277

*α* Cronbach’s alpha, *CR* composite reliability, *FD* factor determinacy, *AVE* average variance extracted

### Reliability

Reliability coefficients and indicators of internal consistency were calculated to assess reliability (i.e., Cronbach’s alpha, Composite Reliability (CR), and Factor Determinacies (FD)). For CR, the larger the coefficient, e.g., ≥ .70, the more stable the factors (Tabachnick and Fidell 2013); for the FD coefficient, the range is from 0 to 1, with 1 being the best value (Muthén and Muthén 2012) (Table 4).

### Concurrent and Criterion-Related Validity

The concurrent validity was investigated with the associations to two other theoretically related constructs, i.e., Internet gaming disorders and social media addiction, based on empirical evidence found in previous studies (Andreassen et al. 2013, 2016; Monacis et al. 2016, 2017b). The criterion validity was assessed between the IDS-15 overall scores and time spent on the Internet. Both validities were performed with bootstrapped correlations with 95% bias-corrected and accelerated (BCa) confidence intervals (Table 5).

**Table 5:** Bootstrapped correlation matrix with 95% BCa confidence interval between the total score of the IDS-15, its four dimensions, BSMAS, IGDS, age of Internet use initiation, and hours of Internet use

	*R*	BCa 95% CI	*R* ^2^
EDEC	.810^**^	.769–.844	.66
WS	.886^**^	.865–.906	.78
IDSR	.796^**^	.758–.830	.63
DISC	.704^**^	.634–.765	.49
BSMAS	.705^**^	.649–.756	.50
IGDS9-SF	.615^**^	.520–.694	.38
Age of Internet use initiation	.065	−.067–.206	.00
Hours of Internet use	.286^**^	.213–.358	.08

*r* Pearson’s coefficient, *R* ^2^ coefficient of determination

**p < .001

### Latent Profile Analyses (LPAs)

**Fig. 2:**
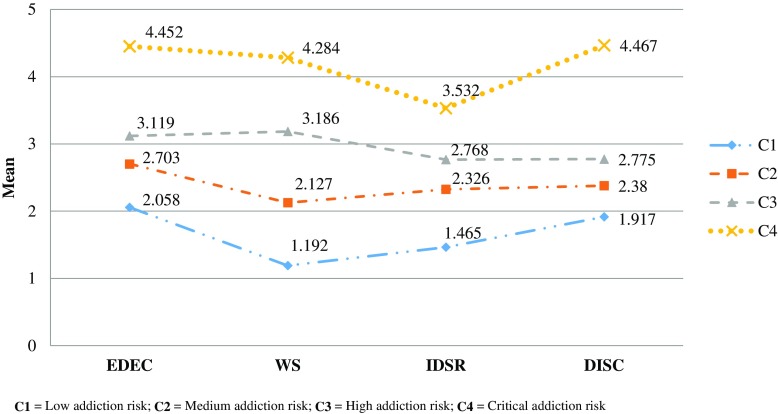
The four-class solution obtained from the LPA. C1 low addiction risk, C2 medium addiction risk, C3 high addiction risk, and C4 critical addiction risk

The fit indices emerging from the LPA analyses demonstrated that the four-class solution was the best, because of the significant level of the *p* value associated with the LMR Test and because the AIC, BIC, and SSABIC values decreased with the addition of more classes (Table 6). A graph of the final four latent classes is shown in Fig. 2. Class 1 was labeled as “low addiction risk” and included individuals (*n* = 137, 30.11%) with mean scores < 2 on all four dimensions of the IDS-15 and with an IDS-15 mean score of 24.55 (SD = 5.03). Class 2, “medium addiction risk,” identified Internet users (*n* = 199, 43.74%) with EDEC, IDSR, and DISC mean scores > 2 and with an IDS-15 mean score of 35.93 (SD = 4.33). Class 3, “high addiction risk,” comprised individuals (*n* = 91, 20.00%) with higher scores on the EDEC and WS dimensions and lower scores on the IDSR and DISC dimensions and an IDS-15 mean score of 44.98(SD = 4.76). Finally, class 4, “critical addiction risk,” included Internet users (*n* = 28, 6.15%) with mean scores markedly high on the EDEC, WS, and DISC dimensions and an IDS-15 mean score of 62.57 (SD = 5.18).

**Table 6:** Goodness-of-fit indices obtained from the three solutions

	AIC	BIC	SSABIC	Entropy	L-M-R test (*p*)	*N* for each class
2 classes	7549.011	7614.936	7564.157	.859	785.239 (.017)	c1 = 318 c2 = 137
3 classes	7051.637	7142.284	7072.463	.881	495.870 (.376)	c1 = 144 c2 = 262 c3 = 49
4 classes	4115.767	4210.534	4137.540	.867	112.042 (.000)	c1 = 137 c2 = 199 c3 = 91 c4 = 28

## Discussion

The aim of the present study was to assess the psychometric properties of the Italian version of the IDS-15 based on the modified criteria for the IGD in the DSM-5 (APA 2013). Consequently, construct validity was examined taking into account the factorial structure and the measurement invariance across gender, as well as the convergent and discriminant validity of the IDS-15 screening tool. Factorial structure analyses indicated that both first- and second-order solutions with four factors yielded satisfactory results. The factor loadings were substantial and statistically significant, thus demonstrating that indicators were adequate reflections of their constructs. The unexpected covariance between the residual errors for items 9 and 10 was theoretically justifiable, because the two items referred to the same dimension (i.e., “impairments” and “dysfunctional self-regulation”). These results corroborated the factorial validity reported by Pontes and Griffiths (2017). The MI across gender groups provided evidence for configural, metric, and strict invariances. In this respect, the four hypothesized factors, the paths between the items and their respective latent factors, and the response ratings in all items were invariant across males and females. Findings from convergent and discriminant validity proved adequate, given that AVE values were higher than the threshold level and MSV values smaller than AVE values. With regard to reliability, the data supported the internal consistency of the IDS-15, demonstrating that the measure was reliable and accurate in assessing IA.

The results from the correlation analyses showed high and positive associations between Internet disorder, Internet gaming disorder, and social media addiction. These findings confirmed previous data (e.g., Andreassen et al. 2013, 2016; Monacis et al. 2017b; Sinatra et al. 2016), thus supporting the conception of Internet addiction as an *umbrella construct* comprising a wide range of online activities (Kuss and Billieux 2017). While the concurrent validity was enhanced, the criterion-related validity was partially warranted, because IA was positively related to time spent on the Internet but unrelated to the age of Internet use initiation, unlike in previous research (Pontes and Griffiths 2015b, 2017; Pontes et al. 2014). This is an issue that should be further investigated.

The results from the LPAs provided four classes of Internet users on the basis of the scores obtained in all four dimensions of the IDS-15. Just below one third of the total sample was classed as having a “low addiction risk” (30.11%) because they exhibited low scores in all dimensions and, therefore, few problems related to Internet usage. Over one third of the Internet users were categorized as “medium addiction risk” (43.74%) because they had problems in the subdomains EDEC, IDSR, and DISC. Here, respondents used the Internet more frequently to escape and dysfunctionally cope with their emotions while also exhibiting difficulties in self-regulation and self-control. When comparing the first class with the second, the scores in all four dimensions indicated similar trends. It is noteworthy that the EDEC dimension yielded the highest scores in both classes. One fifth of the participants belonging to the “high addiction risk” class (20%) were characterized by a higher tendency to escape and dysfunctionally cope with their emotions, as well as experiencing more withdrawal symptoms due to excessive Internet use, and having a lower tendency to experience impairments alongside dysfunctional self-regulation in Internet use and difficulties in Internet-related self-control. The final class was labeled “critical addiction risk” on account of the high scores obtained in all dimensions. However, the subdomain IDSR clearly showed lower scores than the remaining dimensions. That is, the excessive online behaviors appeared to be characterized mainly by the avoidance of negative emotions, the experience of withdrawal symptoms, and the difficulty in controlling Internet usage, and—to a lesser extent—by conflicts and impairments in dysfunctional self-regulation in Internet use.

In comparison with Pontes and Griffiths’ (2017) study, the present research found a further class, here defined as “critical addiction risk,” that may include actual addicts. It is very possible that the individuals belonging to this class were the respondents who were recruited in the gaming halls and may have also been affected by other behavioral addictions. This unexpected result could be a starting point for further investigations with broader clinical samples, thus responding to Pontes and Griffiths’ (2015a, 2017) suggestions for replication of the already identified classes and the establishment of an empirical and clinical cut-off point for the IDS-15.The study is not without its limitations. The sample size was modest (but sufficient for the analysis carried out) and only included a self-selected (i.e., non-representative) convenience sample of young Italian participants. Furthermore, the data were cross-sectional and self-report both of which have methodological shortcomings and well-known biases. Despite these limitations, the present study demonstrated the psychometric robustness of the IDS-15 (i.e., validity and reliability) based on the modified IGD criteria. Future studies should be carried out in cross-cultural research contexts in order to expand and generalize the present findings.
